# Advances in Synthetic Polymer Membranes for Guided Bone Regeneration in Dental Implants: A Scoping Review

**DOI:** 10.3390/jfb16050149

**Published:** 2025-04-22

**Authors:** Belén Lima-Sánchez, María Baus-Domínguez, María-Angeles Serrera-Figallo, Daniel Torres-Lagares

**Affiliations:** Department of Stomatology, Faculty of Odontology, University of Seville, C/Avicena S/N, 41009 Seville, Spain; bellimsan@alum.us.es (B.L.-S.); maserrera@us.es (M.-A.S.-F.)

**Keywords:** bone regeneration, membrane, polymeric materials, implant dentistry

## Abstract

Background: Different approaches are proposed for bone volume gain in the case of atrophic alveolar ridges, with guided bone regeneration (GBR) and guided tissue regeneration (GTR) being the most used techniques. These techniques require the placement of barrier membranes, which is the main element of the bone growth strategy, among which there is a wide range depending on their origin or degradation. This literature review aims to provide an update on the latest advances in polymeric membranes of synthetic origin currently used in bone regeneration. Materials and Methods: Two bibliographic searches were carried out in the PubMed (MEDLINE) and Scopus databases using a search strategy in which inclusion and exclusion criteria were applied. Results: For the selection of articles, the PRISMA guide flow chart was followed, and after a selection process, 11 articles were analyzed based on the characteristics of the marketed membranes and the results obtained after their use. Conclusions: It can be concluded that polymeric membranes play a fundamental role in guided bone regeneration, providing an effective barrier that facilitates bone growth and improves the success of dental implantology treatments.

## 1. Introduction

Rehabilitation of edentulous spaces using dental implants is an increasingly frequent treatment option in dental clinics. To correctly place them in the most favorable position and thus improve their long-term prognosis, it is common to need an increase in bone volume before or simultaneously with the placement of implants [1]. This is because the alveolar ridge resorbs after tooth extraction, although trauma or infection can also play a role [2]. The formation and preservation of the alveolar process depends on the presence of teeth since the forces generated during mastication are transmitted from the crown of the tooth to the alveolar bone tissue, where the loads are dispersed, maintaining a balance in the remodeling of the process. If tooth loss occurs, resorption of the alveolar processes is inevitable since the application of forces on an edentulous alveolar process causes bone resorption in the area, changing the initial architecture of the bone [3]. The most frequent occurrence is the loss of volume at the horizontal level compared to the loss of height at the vertical level due to the remodeling pattern [1,2].

For bone volume gain in the case of atrophic alveolar ridges, different approaches are proposed; on the one hand, there is the option of guided tissue regeneration (GTR), which was established to redirect periodontal ligament fibroblasts and promote periodontal regeneration in situations of chronic periodontitis [4]. On the other hand, there is guided bone regeneration (GBR), which modulates the promotion of bone neoformation in bone defects through osteogenesis, osteoconduction, or osteoinduction, restoring the structural and functional characteristics of bone tissue [1,4,5]. The same types of barrier membranes can be used for both techniques; however, in GTR, collagen membranes are more commonly employed, as the procedure requires less membrane rigidity compared to GBR. In GTR, reinforcement or the use of osteosynthesis screws is generally unnecessary, clot stability is less critical, and the overall regeneration period is shorter, given that soft tissue regenerates faster than bone tissue [5]. These techniques force the placement of mechanical barriers (membranes), which is the elementary principle of bone growth strategy, whose primary function is to guide the regeneration of hard and soft tissues, preventing the invagination of epithelial and connective tissue into the bone space [2,5,6], achieving the protection of blood clots and the increase in surrounding tissue, providing the necessary space to bone-forming cells for regeneration and vascularization [2]. The most common treatment approach is guided bone regeneration, which refers to the augmentation of the alveolar ridge itself and includes cell differentiation, cell proliferation, and induction and/or conduction of tissue formation [7].

The design of an ideal GBR membrane must have several properties [8,9]:

- Biocompatibility: no damage to surrounding tissue or the healing process.
- Cell occlusion: prevents the invasion of non-osteogenic cells into the bone defect.
- Easy handling: it cannot be too rigid without losing the function of space maintenance.
- Bioactivation properties promote wound healing and tissue integration.

It is considered acceptable for barrier membranes to require 4 to 6 weeks of rest in cases of periodontal tissue regeneration and 16 to 24 weeks for bone regeneration [8].

### 1.1. Rankin

Choosing an appropriate barrier membrane is a critical determinant of success in guided bone regeneration (GBR) [10]. Currently, membranes are classified based on their origin (allogeneic, xenogeneic, or alloplastic) or their degradation profile (resorbable or non-resorbable) [5].

According to their origin, membranes may be naturally derived—typically biopolymers, with collagen being the most widely used—or synthetically derived. Synthetic membranes include materials such as polycaprolactone (PCL), polylactic acid (PLA), polyglycolic acid (PGA), their copolymers poly (lactic-co-glycolic acid) (PLGA), polyether ether ketone (PEEK), and expanded polytetrafluoroethylene (ePTFE). Recent developments have expanded the category of non-resorbable synthetic membranes to include dense polytetrafluoroethylene (d-PTFE), titanium-reinforced PTFE, and titanium mesh. The selection of membrane type depends on factors such as mechanical strength, biocompatibility, and the capacity to support cellular and bone regeneration [5,11,12,13].

Resorbable membranes, composed almost exclusively of either natural or synthetic polymers, are commonly used in GBR. Among them, collagen-based membranes are the most frequently selected due to their commercial availability, excellent biocompatibility, and predictable clinical outcomes [12]. These membranes are cost-effective and undergo natural degradation, eliminating the need for a second surgical procedure and thereby reducing patient discomfort and complication rates. However, their lower mechanical strength and rapid degradation may compromise regenerative outcomes [11,12].

In contrast, non-resorbable membranes—typically composed of synthetic polymers, metals, or their composites—offer superior mechanical strength [12]. Nonetheless, they require surgical removal, increasing patient morbidity [13]. Their inherent rigidity can complicate handling, and their porous structure may facilitate bacterial colonization, making membrane exposure a risk factor for compromised outcomes compared to resorbable alternatives [12,13].

The degradation of resorbable membranes occurs via hydrolysis or enzymatic activity over a span of weeks to months [12]. This rate can be modulated by altering polymer composition, molecular weight, and crystallinity. Natural membranes, being more hydrophilic, tend to absorb fluids and degrade more rapidly than synthetic ones [12]. Synthetic resorbable membranes are designed to retain structural integrity throughout the regenerative phase, after which they are resorbed by the host tissue. Their degradation is influenced by polymer type, structural characteristics, physiological conditions, and manufacturing techniques [11,12].

### 1.2. Polymeric Membranes

Non-resorbable membranes include expanded polytetrafluoroethylene (e-PTFE), high-density polytetrafluoroethylene (d-PTFE), titanium-reinforced PTFE, and titanium mesh [11,14]. PTFE is considered the gold standard due to its high predictability compared to resorbable membranes. However, it presents disadvantages such as low cell adhesiveness and a complete lack of bonding capacity with bone tissue, which prevents osseointegration [15].

Natural biodegradable polymers play a key role as scaffold materials in guided bone regeneration. They offer several advantages, including biodegradability, biocompatibility, strong cell adhesion, and a favorable environment for cell growth. Nonetheless, they also present limitations, such as restricted absorption capacity, poor mechanical strength, immunogenicity issues, and the risk of contamination with pathogenic impurities [13].

In addition to natural polymers, synthetic resorbable membranes have been developed using materials such as polyglycolic acid (PGA), polycaprolactone (PCL), and poly (lactic-co-glycolic acid) (PLGA), among others. These synthetic polymers allow for precise control over composition, scalable production, and the customization of key properties—such as mechanical strength, porosity, and degradation rate—to meet specific clinical needs. However, their limitations include potential immunogenicity and toxicity, hydrophobicity, and poor cell binding capacity, which can impair cell adhesion [13].

Based on the current literature, this review aims to provide an updated overview of recent advances in synthetic polymeric membranes for bone regeneration.

Table 1 presents a comparative overview of the general characteristics of the main polymeric membranes used in guided bone regeneration. It includes a brief description of each membrane type, highlighting their composition, representative trade names, key biological and mechanical properties, and whether they undergo any specific surface treatment or modification to enhance their clinical performance. This summary aims to facilitate a clearer understanding of the differences among available options and support clinicians in selecting the most appropriate membrane based on case-specific requirements.

## 2. Materials and Methods

### 2.1. Search and Data Mining Strategy

For this literature review, a search was performed using the PubMed database (MEDLINE) and Scopus as the primary sources for obtaining published articles on guided bone regeneration using synthetic polymeric barrier membranes. The following search strategy was followed in PubMed ((“Membranes, Artificial”[MeSH] OR “Polymers”[MeSH] OR “Biocompatible Materials”[MeSH]) AND (“Polylactic Acid”[MeSH] OR “Polyglycolic Acid”[MeSH] OR “Polylactic-Co-Glycolic Acid”[MeSH] OR “Polytetrafluoroethylene”[MeSH]) AND (“Bone Regeneration”[MeSH] OR “Guided Tissue Regeneration”[MeSH] OR “Dental Implants”[MeSH])) NOT (“Collagen”[MeSH]) and the following in Scopus (Membranes AND artificial OR Polymers OR Biocompatible Materials AND Polylactic Acid OR Polyglycolic Acid OR Polylactic-Co-Glycolic Acid OR Polytetrafluoroethylene AND Bone Regeneration OR Guided Tissue Regeneration OR Dental Implants NOT Collagen)

Only publications written in English and published in the last 10 years (2014–2024 inclusive) were selected. This range of the last 10 years allows us to obtain a broader context and long-term trends regarding the use of synthetic membranes, greater availability of relevant data and studies, and a broader scope in the evolution of the technology and methodologies employed. The reference lists of previous reviews and included studies were analyzed after electronic screening to find relevant missing articles.

### 2.2. Inclusion and Exclusion Criteria

The inclusion criteria considered were randomized controlled trials, nonrandomized controlled trials, cohort studies, case–control studies, case series, and articles published in the last 10 years. The minimum sample size taken into account was 5 patients/5 surgical areas.

The exclusion criteria were animal studies, in vitro studies, reviews or articles published in languages other than English, and studies evaluating bone gain using collagen membranes.

## 3. Results

### 3.1. Search Strategy Results

The electronic search was carried out in October 2024 and yielded 1292 articles. After applying filters, 615 articles were obtained. The filters applied were publication date within the last 10 years, text availability, and article type. After reading the titles and/or abstracts, 45 articles were selected. The full texts of the 45 selected articles were reviewed to determine the inclusion criteria. Thirty-four were excluded after full reading, and eight articles were included in the final selection.

The search in the Scopus database yielded 1422 articles, which, after applying filters, were reduced to 591. Duplicates (125 articles) were eliminated, and 53 were selected after reading the titles and/or abstracts. Fifty articles were excluded after full reading, and three were finally included.

**Figure 1:**
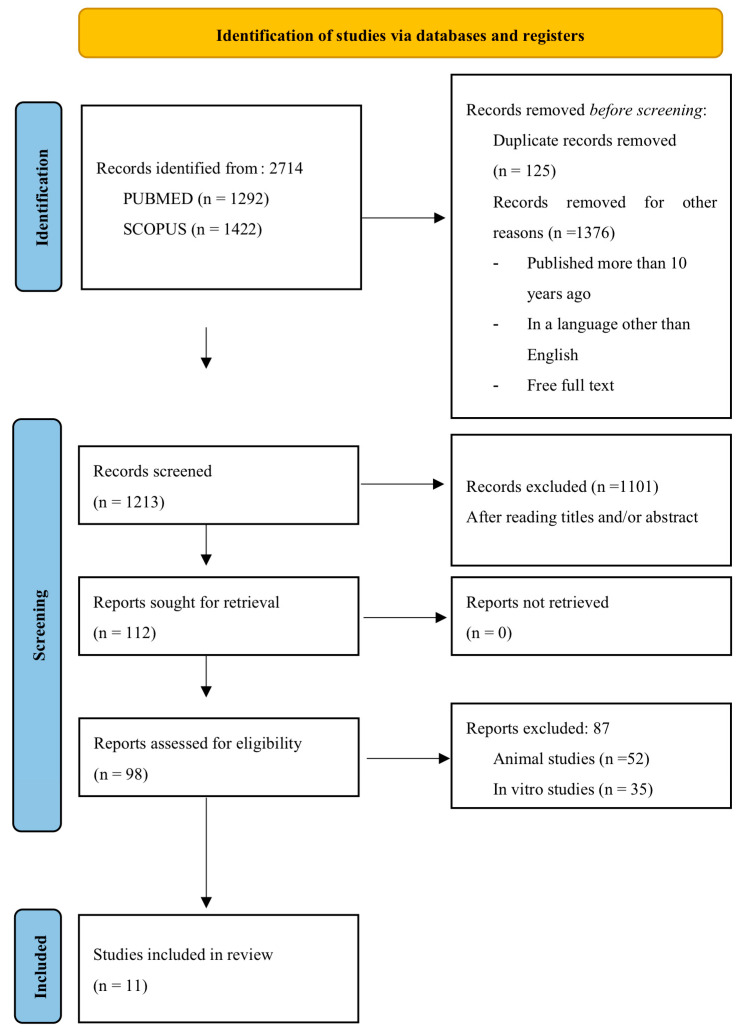
PRISMA flowchart for study selection and assessment.

Despite the extensive search and the initial retrieval of a considerable number of articles, many of them did not meet the inclusion criteria. For example, many studies were conducted on animals, or the follow-up period did not meet the expected standards. Synthetic polymer membranes are not as widely used as collagen or PTFE membranes, which is why there is not a large enough number to expand the final selection. Figure 1 describes the flowchart of identifying items selected from a total search based on PRISMA recommendations.

### 3.2. Summary of the Main Findings of the Search Strategy

Below is a detailed account of the main findings of the articles included for review (Table 2).

## 4. Discussion

### 4.1. Main Findings

The main objective of guided bone regeneration is to create sufficient bone volume to support the patient’s functional and esthetic results. That is why among the key factors for the success of the treatment are the properties of the biomaterials used in the surgery, thus involving barrier membranes, which are the central element in the bone gain strategy [2,5,14].

Several findings can be highlighted based on the articles included in the review and their implementation in regenerating alveolar bone defects. Non-resorbable membranes have been accepted as the gold standard since their inception, despite presenting many drawbacks, as described by Kusirisin et al. (2023) [17], the need for additional surgery for removal, increased patient morbidity, and high rates of membrane exposure due to their highly porous structural design. On the other hand, Palkovics et al. (2022) [16] advocate their use in situations of vertical ridge augmentation. They advocate that these conditions can present challenges due to compromised soft and hard tissue situations and the complexity of flap management. Therefore, they highly recommend the use of non-resorbable membranes for space maintenance. They present, as an advantage, its semi-ductile structure that can be molded and can maintain its shape, thus creating an isolated and safe space. Cucchi et al. (2023) [18] present a comparative study highlighting the clinical success of non-resorbable membranes in vertical ridge augmentation due to several clinical factors that support the above.

Currently, collagen-resorbable membranes are the most widely used in clinical practice due to their excellent biocompatibility and regenerative capacity, similar to non-resorbable ones, thus avoiding their drawbacks [14]. Even so, collagen membranes also present certain disadvantages, such as the risk of transmitting diseases, loss of space maintenance capacity after wetting, higher cost, and rapid biodegradation, in addition to inducing an insufficient result when the membrane is exposed, partly due to bacterial infection derived from the presence of pores large enough for bacterial infiltration [13,15,17]. To overcome these disadvantages of collagen membranes, synthetic resorbable membranes are developed, which can adjust their properties as an alternative barrier membrane in an infinite variety of well-established shapes and structures [14]. Ogata et al. (2022) [24] describe the advantages of synthetic biodegradable materials as the absence of risk of transmission of unknown pathogenic material, thus guaranteeing stable quality, and their usefulness as a protective valuable membrane for guided bone regeneration as they do not require subsequent surgical invasion for removal. In contrast, the study published by Shido et al. (2023) [22] shows several disadvantages in using synthetic polymers as a mechanical barrier, specifically with poly (L-lactide-coprolactone) or P(LA/CL) membranes. First, they present an unpredictable biodegradability; the biodegradation period of the membrane being too short would induce insufficient bone cement. At the same time, a prolonged degradation would require its removal using a surgical procedure. Another disadvantage mentioned is the unpredictable resorption of augmented bone after bone augmentation surgery, which makes it difficult to predict the amount of augmented bone absorbed after guided bone regeneration [22].

### 4.2. Comparison of Resorbable and Non-Resorbable Membranes

#### 4.2.1. Biocompatibility and Mechanisms of Action

The primary function of membranes is to inhibit the migration of rapidly growing connective tissue into the defect and promote hard tissue growth [26]. Barrier membranes must have good biocompatibility to achieve the desired tissue regeneration. This requires that they do not negatively affect the prognosis of the patient’s peripheral cellular tissue, bone defect area, and general health. If the membrane is resorbable, it must be able to degrade or integrate into host tissues, reducing the potential incompatibility caused by the barrier membrane [10]. The surface of synthetic resorbable membranes is microporous, which allows tissue fluid to pass through it but, in turn, prevents connective tissue and epithelial cells from passing through, thus providing reasonable protection of the space for new bone formation. They have a two-sided structure: a smooth one oriented towards the soft tissue, which promotes good healing, and a rough one directed towards the bone tissue, leading to optimal bone healing [25].

Non-resorbable membranes, such as PTFE membranes, which are widely used mainly due to their good biocompatibility, are derived from their two-part structure. On the one hand, the open microstructure portion promotes the ingrowth of collagen fibrils on its surface, improving the stability of the membrane and allowing the diffusion of nutrients through the pores; on the other hand, the occlusive portion is relatively impermeable to fluids and cells and blocks the migration of soft tissue cells into the area of bone growth [27].

Bioactivity in guided bone regeneration is reflected in the osteogenic capacity of barrier membranes. These membranes provide a local bone defect environment that favors osteoblast regeneration and differentiation. Clinical research is focused on improving this bioactivity and osteoinduction by designing an improved membrane structure [10,19].

#### 4.2.2. Mechanical Resistance and Clinical Management

Non-resorbable membranes, specifically PTFE membranes, were the first to be used as barrier membrane material in guided bone regeneration. They have good biocompatibility, inertness, and resistance to decomposition [19,21]. Another type of non-resorbable membrane is titanium meshes, which, given their good flexibility, can be adapted to various bone anomalies. However, the stiffness and sharp edges from trimming and contouring can make them hard to handle, irritate the mucosa, and increase the risk of exposing the membrane [19,22]. The stiffness exhibited by non-resorbable membranes, which may be increased with simultaneous implant placement, may be compromised by masticatory function [21,22].

Synthetic resorbable membranes, such as PLA and PGLA, can be difficult to manipulate during surgical procedures due to their stiffness. However, the use of softeners has helped to reduce this issue. PCL membranes, while offering good mechanical properties, have limitations in terms of their ability to withstand mechanical loads [10,21]. Young’s modulus is used to express the mechanical properties of barrier membranes. A low Young’s modulus corresponds to a higher flexibility than membranes with a high Young’s modulus. In a study published by Caballé-Serrano et al. (2019) [5], an analysis shows that Young’s modulus is tested in different barrier membranes using a table comparing the initial and final stiffness. Among the membranes that have a high stiffness are those of PTFE, but the curious thing is that their Young’s modulus is comparable and similar to that of resorbable synthetic membranes of polycaprolactone (PCL) and polylactic-glycolic acid (PGA) (high Young’s modulus).

Regarding their clinical management, non-resorbable membranes present the need for additional surgery to remove the membrane, which increases the risk of exposing the newly regenerated bone. Choosing the timing of membrane removal wisely is essential, as early removal can lead to resorption of the newly regenerated bone and late removal increases the risks of bacterial contamination and infection [13,22]. Resorbable synthetic membranes facilitate clinical management by improving stiffness by adding softeners. They also have the advantage of not requiring a second surgery for their removal. However, it is essential to know how to choose which type of membrane we are interested in according to its degradation time [10]. PLA or PGLA membranes have a complete degradation time of 3 to 4 weeks, thus avoiding interrupting the healing process. PCL membranes, on the other hand, have a long resorption time (approximately more than 24 months), which is considered a significant limitation when choosing them [27].

### 4.3. Impact of Technological Advances

#### 4.3.1. Modern Synthetic Membranes

Previous studies have shown that pore size determines synthetic non-resorbable PTFE membranes’ regenerative capacity and clinical management. A larger pore size could improve the biological effects, but the contact with the membrane is too high, making membrane removal difficult. Moreover, its exposure could lead to bacterial infection, decreasing complex bone formation and postoperative complications [10,23]. Because of these complications caused by membrane exposure, the PTFE membrane is gradually replaced with an expanded PTFE or e-PTFE membrane [10]. These membranes have a double-layer structure with pores between 5 and 30 microns, which could allow better transmembrane delivery of nutrients and improved graft maturation [13,16].

Later, a dense PFE or e-PTFE membrane with an even smaller pore size (0.2 microns) was created [10,16]. It serves as a more efficient barrier against bacterial penetration and epithelial growth [17]. On the other hand, the blood supply to this area is also limited because of the limited porosity [10,16].

Titanium-reinforced e-PTFE and d-PTFE membranes were produced to address the lack of mechanical stiffness in the initial membranes. Still, the requirement for membrane removal surgery is considered the most significant disadvantage [13].

In a study published by Palkovics et al. (2022) [16], the effects of PTFE exposure during vertical bone regeneration are evaluated. It is observed that when a d-PTFE membrane is exposed, there is less negative impact on clinical outcomes than the results with e-PTFE membranes. Therefore, it can be highlighted that the dense structure and small pore size of the d-PTFE membrane may have contributed to these positive effects.

#### 4.3.2. Special Treatments and Bioactive Properties

It should be considered that, in numerous cases, guided bone and tissue regeneration in periodontal procedures faces obstacles due to contamination and infection at the healing site [24,25,26]. Systemic administration of antibiotics is effective, but very high doses and long-term use are needed, which can create new resistant strains [28]. This is why research has focused on optimizing the functionality of these membranes. It has been proposed that antibacterial agents be incorporated to prevent bacterial contamination during surgery or in the healing period, especially if the membrane is exposed to the oral cavity [10,24].

Although the natural collagen membrane is the most widely used biomaterial as an antimicrobial carrier material, synthetic polymers are also widely used [11]. Among the resorbable ones, we find PLA, PGA, PCL, or combinations between them, and among the non-resorbable ones, there are two that are frequently used: one is expanded polytetrafluoroethylene (ePTFE), and the other, a new polymer based on hydroxy ethylene methyl methacrylate (HEMA-MMA) [24,25,26].

The most frequently used antibiotics in barrier membranes are tetracyclines, which are broad-spectrum antibiotics capable of fighting most of the responsible bacteria. Among the most frequent are minocycline, but especially doxycycline [24,25]. They inhibit protein synthesis in bacteria and have been shown to have a prolonged shelf life. They have anti-collagenase properties and are well absorbed by bone due to their calcium-chelating effect [11]. Metronidazole is the second most commonly used antibiotic, which alters nucleic acid synthesis in bacteria [28,29].

Several techniques are used to fabricate these antibiotic-loaded barrier membranes with controlled release function, including phase separation, solvent evaporation, and electrospinning, the latter being the most widely employed [28,29,30,31].

Other proposals include the development of bioactive barrier membranes to control growth factors with different biological activities that play a role in the various stages of bone healing and mimic the process of natural osteogenesis by incorporating bone morphogenetic protein (MBP) [10,27], or the incorporation of zinc or zinc alloys into GBR membranes. Zinc participates in the growth and mineralization of bone tissue and inhibits bone resorption by inhibiting the formation of osteoclast-like cells in bone marrow cells [10].

### 4.4. Complications and Risk Factors

As mentioned above, the most frequent complication of GBR is membrane exposure, especially when the membrane used is polytetrafluoroethylene (PTFE). Depending on the size of the exposure, they can be classified into different classes [15,30]:

- Class I: Small membrane exposure (<3 mm) without purulent exudate.
- Class II: Considerable membrane exposure (>3 mm) without purulent exudate.
- Class III: Exposure of the membrane with purulent exudate.
- Class IV: Abscess formation without exposure of the membrane.

Palkovics et al. (2022) [16] propose a series of guidelines depending on what type of exposure has occurred. In the case of class I exposure, the exposed part of the membrane is removed, and secondary epithelialization covers the whole area after one week. In the case of class II exposure, after removal of the exposed part, the membrane is kept in place for at least 6 weeks (it is removed between weeks 6 and 12); in the meantime, patients are regularly scheduled for irrigation. Regarding the data obtained in their study, the exposure of the d-PTFE membrane seemed to have a less negative impact on surgical outcomes than the data obtained on e-PTFE membranes, mainly due to the pore size of the new-generation d-PTFE membrane. On the other hand, Windisch et al. (2021) [15] presented only one case of d-PTFE membrane exposure, where the membrane is removed and replaced with a collagen membrane 6 weeks after surgery, resulting in adequate augmentation for subsequent implant placement by compensating for the loss of soft tissue with the placement of a connective tissue graft.

Arunjaroensuk et al. (2018) [23] refer to rigidity and difficult adaptation as risk factors for membrane exposure in PLA resorbable membranes. Therefore, previous modeling is recommended before adapting them to the regeneration site, and the membrane should first be soaked in a physiological saline solution to increase its softness. In their article, Li et al. (2023) [25] mention this recommendation by Arunjaroensuk et al., since PLA resorbable membranes can make covering the graft more difficult due to their hardness and tenacity. In addition, they mention the lack of reactive functional groups present in PLA membranes, which results in insufficient hydrophilicity and poor cell adhesion.

Finally, Maiorana et al. (2021) [21] show as an essential aspect in their study the rate of complications obtained, where premature exposure to non-resorbable membranes, which can compromise the final regeneration result, is considered the most common complication but only occurred in cases where titanium mesh was used. It is speculated that the stiffness and the sharp edges of the titanium mesh may have induced mechanical irritation to the mucosal flaps, thus increasing the risk of soft tissue dehiscence with consequent exposure. As a possible solution, the placement of an additional protective collagen membrane towards the soft tissues against the irregularities of the titanium mesh is assessed, thus reducing the risk of flap dehiscence, at least during the early stages of healing.

Membrane exposure is a significant complication in GBR procedures, and its consequences vary depending on the type of membrane used [15,20]. For non-resorbable membranes such as e-PTFE and titanium meshes, exposure may lead to bacterial colonization, tissue inflammation, graft disintegration, and the need for premature removal, resulting in reduced hard tissue formation [15,20]. New-generation d-PTFE membranes appear to show fewer negative outcomes in cases of exposure compared to e-PTFE membranes [16].

In the case of resorbable membranes, although they are considered safe, exposure can also have negative consequences. For example, resorbable collagen membranes, when exposed, can increase the risk of immune responses, transmission of infectious agents, and faster degradation, compromising the ability to maintain the necessary space for bone regeneration [23].

### 4.5. Clinical Applications and Results

#### 4.5.1. Efficacy in Different Clinical Scenarios

The main objective of guided bone regeneration is bone gain in edentulous sites using resorbable and non-resorbable barrier membranes [15]. It is interesting to analyze the results regarding bone gain in both vertical regeneration and horizontal regeneration to assess the efficacy of each in different clinical scenarios.

First, Arunjaroensuk et al. (2018) [23] analyzed the efficacy of a synthetic PLA membrane compared to a resorbable collagen membrane, giving no statistically significant results between the control and test group (*p* > 0.05) and concluding that the amount of stable augmented bone obtained with a PLA membrane was similar to that obtained by collagen membrane regeneration [28]. The same comparison was made in the study published by Li et al. (2023) [25]. The horizontal bone gain in the experimental (PLA) and control (collagen) groups was 2.99 ± 0.76 mm and 2.83 ± 0.81 mm, respectively. At both 6 months and 36 months postoperatively, the changes at the vestibular level in both groups were not statistically significant, with *p* = 0.281 at 6 months and *p* = 0.71 at 36 months. Therefore, it is concluded that PLA membrane is comparable to collagen membrane in terms of efficacy and safety and can be used clinically as a barrier membrane to guide bone regeneration [20].

Continuing with the analysis of bone gain in resorbable synthetic membranes, Kusirisin et al. (2023) [17] compared the PCL membrane with the collagen membrane. The bone gain values between both study groups were not statistically significant in the three measurement locations (on the platform, at 2 mm, and 4 mm), as in all *p* > 0.05. Therefore, it can be concluded that a bilayer PCL membrane could be an alternative for GBR with simultaneous implant placement with dehisced implant defects.

On the other hand, Shido et al. (2023) [22] compared a collagen membrane with a P(LA/CL) membrane. The increased width was measured at 1, 3, and 6 mm from the implant neck in both groups. No significant differences were observed between the P(LA/CL) membrane and the collagen membrane concerning bone augmentation potential, thus concluding that the P(LA/CL) membrane could be used as a safe and effective membrane in GBR. This study showed that the resorption rate of the augmented bone volume varied widely in both the collagen and P(LA/CL) groups, indicating the difficulty in predicting the amount of bone absorbed after GBR. Even so, the P(LA/CL) membrane was shown to have bone augmentation potential similar to the collagen membrane in GBR. P(LA/CL) is a synthetic polymer that can be used as a safe and effective membrane in GBR. In support of the conclusions of the previous article, Ogata et al. (2022) [24] analyzed the P(LA/CL) membrane as an effective alternative for guided bone regeneration, demonstrating that the use of this membrane did not cause any irreversible adverse effects and showed sufficient performance to regenerate alveolar bone as a GBR membrane.

Regarding non-resorbable membranes, Arbab et al. (2016) [19] performed a randomized clinical trial where bone gain results obtained with a PTFE membrane and collagen resorbable membrane were compared, and no significant differences were observed between a collagen membrane and a PTFE membrane in terms of ridge width or height dimensions or bone and histological composition. No statistically significant differences were found between the two membranes regarding increased bone ridge resorption. From a clinical point of view, this means that both membranes work well in preserving ridge dimensions. The percentage of vital bone for the collagen group was 24 ± 16% versus 30 ± 23% for the PTFE group. Thus, both membranes favor substantial bone formation. In 2021, Windisch et al. [15] performed a study where vertical and horizontal gain with a d-PTFE membrane was measured both in GBR simultaneous to implant placement, being 3.2 ± 1.9 mm and 6.5 ± 0.5 mm, respectively, and in GBR by stages, being 4.5 ± 2.2 mm and 8.7 ± 2.3 mm, respectively. From a clinical point of view, these results can ensure predictable crestal bone stability, which is one of the essential criteria for short- and long-term clinical success. Palkovics et al. (2022) [16] also analyzed the results obtained using a d-PTFE membrane, obtaining a vertical gain of 3.8 ± 0.54 and a horizontal gain of 5.75 ± 0.87. They concluded that the new-generation d-PTFE membrane had a less negative impact on clinical outcomes than published data on e-PTFE membranes. They highlight that the dense structure and small pore size of the new-generation d-PTFE membrane may have contributed to the positive results.

Finally, comparisons were made between d-PTFE membranes and titanium meshes. Maiorana et al. (2021) [21] obtained results of vertical bone gain, with a mean of 4.2 and 1.5 mm in the d-PTFE and TM groups, respectively, with a *p* = 0.06. Mean mineralized tissue of 48.28 and 35.54% in d-PTFE and TM, respectively, with a *p* = 0.51. Therefore, they concluded that the vertical gain was higher in d-PTFE, although there was no statistically significant difference. The same conclusion was reached in a study by Cucchi et al. (2023) [18], in which the titanium mesh was covered with a resorbable collagen membrane. The two study groups observed a statistically significant difference in interproximal bone peaks (IBL). According to these findings, using a titanium mesh covered with a cross-linked collagen membrane yielded more favorable results than a PTFE membrane. Importantly, non-resorbable membranes achieve a thinner pseudo-periosteal layer above the newly formed bone compared to titanium mesh covered with collagen membrane. Overall, PTFE membranes have been shown to produce more significant bone augmentation, less pseudo-periosteum formation, and more substantial bone resorption. In contrast, titanium meshes showed a bone reduction gain associated with thicker pseudo-periosteum and less bone reduction over time [18].

#### 4.5.2. Subgroups and Customized Strategies

Once the selected articles have been analyzed, recommendations can be made regarding the type of membrane to use based on the bone defect and the patient’s needs. Non-resorbable polymeric membranes made of d-PTFE and e-PTFE are particularly suitable for guided bone regeneration in complex vertical defects and areas subjected to high mechanical load, due to their high stability, excellent space maintenance, and low bacterial permeability. However, they carry the risk of a higher likelihood of membrane exposure [15,23]. Titanium-reinforced d-PTFE is recommended for procedures that require significant vertical regeneration [15,23]. In contrast, titanium meshes are preferred for complex defects that need large-volume bone regeneration, thanks to their excellent ability to maintain space, moldability, and high mechanical strength. However, they present a higher risk of exposure and postoperative complications. Covering with collagen membranes is recommended to minimize risks [21].

Resorbable synthetic polymeric membranes are suggested for use in areas of low to moderate mechanical load or in patients who prefer to avoid animal products. They have a lower space-holding capacity than non-resorbable membranes and are suitable for horizontal defects [15,32].

### 4.6. Limitations of the Study and Future Perspectives

#### Limitations of the Review

The current review on polymeric membranes for guided bone regeneration presents several methodological limitations in the articles analyzed. The sample size was small as many studies included limited patients or surgical areas, decreasing the results’ generalizability. Follow-up times varied widely, from a few months to several years, complicating the direct comparison of results on efficacy and complications. In addition, there is much diversity in the study designs; prospective studies, randomized clinical trials, and case series were analyzed, which introduces methodological heterogeneity. Finally, complications are presented that, although membrane exposures are mentioned, show a limited focus on related issues, with not all studies providing a detailed analysis of the complications associated with each type of membrane.

## 5. Conclusions

Based on the results obtained and after an exhaustive analysis of the selected articles, it can be concluded that polymeric membranes play a fundamental role in guided bone regeneration, providing an effective barrier that facilitates bone growth and improves the success of dental implantology treatments. Their versatility allows a balance between biocompatibility, degradation control, and mechanical strength, which is essential for maintaining the regenerative space. Resorbable and non-resorbable synthetic polymer membranes have proven viable alternatives to collagen membranes, offering options tailored to specific clinical needs. However, significant challenges remain related to stiffness, unpredictable biodegradability, and membrane exposure, which may compromise clinical outcomes.

To advance these solutions’ efficacy, fostering innovation in membrane design is imperative. The integration of new technologies, such as artificial intelligence, to customize physical and chemical properties; the exploration of advanced polymers with greater biological functionality; and the incorporation of bioactive agents represent the future of this area. In addition, clinical studies with larger sample sizes and prolonged follow-ups are required to consolidate evidence and ensure long-term safety and effectiveness. This continuous improvement approach will overcome limitations and establish new frontiers in guided bone regeneration, optimizing functional outcomes and patient experience.

## 6. Future Research Directions

Thanks to the impact of new technologies and their continuous development, future research directions can be proposed, such as integrating artificial intelligence (AI) in membrane design, which can optimize the customization of mechanical properties, controlled degradation, and pore structure of membranes to improve their performance. Novel polymers that provide better biocompatibility, cell adhesion, and tunable mechanical properties while avoiding immunogenicity and hydrophilicity issues could be explored. Long-term clinical studies with larger sample sizes are needed, along with more robust investigations, to validate preliminary findings and improve the evidence on the performance of synthetic polymeric membranes.

This approach will overcome limitations and maximize the potential of membranes in guided bone regeneration, favoring more effective and less invasive treatments.

## Figures and Tables

**Table 1:** General characteristics of the different polymeric membranes, providing a brief description of each, including trade names, biological and mechanical properties, and whether they undergo any special treatment.

Membrane Type	Description	Trade Names	Biological Characteristics	Mechanical Characteristics	Special Treatment
**e-PTFE**	Non-resorbable membrane widely used since the 1990s; effectively maintains space for bone regeneration.	Gore-Tex^®^	High biocompatibility; effective barrier against soft tissue invasion.	High stiffness and structural integrity during regeneration.	No special treatment; requires careful handling due to infection risk if exposed [11,14,15].
**d-PTFE**	Variant of e-PTFE with reduced porosity, offering lower susceptibility to bacterial contamination.	Cytoplast^®^	Low risk of infection; effective graft protection even in case of exposure.	High density; stable and easy to retrieve.	No special treatment required; suitable for open healing [11,13,14].
**PLA**	Synthetic, resorbable membrane derived from lactic acid.	Epi-Guide^®^	Biodegradable, with adequate biocompatibility and low immunogenicity.	Moderate mechanical strength; may require reinforcement depending on clinical use.	No special treatment typically needed [12,13].
**PGA**	Resorbable membrane similar to PLA but with a faster degradation rate.	—	High biocompatibility; rapid degradation through hydrolysis.	Low stiffness; susceptible to deformation under load.	No special treatment required [12,13].
**PLGA**	Copolymer combining PLA and PGA, enabling tailored degradation kinetics.	Resolut Adapt^®^ Guidor^®^	Good biocompatibility; adjustable degradation profile.	Balanced flexibility and strength; greater stability than PGA.	No special treatment required [12,13].
**Membranes with SiO_2_**	Electrospun membranes incorporating silica nanoparticles to enhance osteoconduction.	—	Promote osteoconductivity and angiogenesis.	Improved mechanical strength and integration due to nanoparticles.	May be functionalized with zinc or antimicrobials (e.g., doxycycline) [13].
**PCL**	Synthetic, slowly resorbable polymer membrane.	Guidor^®^ Cytoplast^®^	Biocompatible with good integration; very slow degradation rate.	High flexibility; good mechanical resistance.	Can be blended with chitosan or other additives to enhance properties [12,13].

— No commercial house has been found.

**Table 2:** Overview of the included studies. The table includes information regarding the type of membrane used in the test group (TG) and the control group (CG), follow-up time, number of patients or areas treated, bone gain results, and any complications that occurred in each case.

Author	Type of Study	Membrane Used		Follow-Up Time	Number of Patients/ Surgical Areas	Results Obtained		Complications
		Test Group (TG)	Control Group (CG)			Vertical (mm)	Horizontal (mm)	
Windisch et al., 2021 [15]	Prospective study	d-PTFE reinforced with Ti	No	9 months	19 patients/ 24 surgical areas	TG in stages: 4.5 ± 2.2 Simultaneous TG: 3.2 ± 1.9	TG in stages: 8.7 ± 2.3 Simultaneous TG: 6.5 ± 0. 5	1 membrane exposure
Palkovics et al., 2022 [16]	Prospective study	d-PTFE not reinforced	No	9 months	8 patients/ 8 surgical areas	TG: 3.80 ± 0.54	TG: 5.75 ± 0.87	4 membrane exposures
Kusirisin et al., 2022 [17]	Randomized clinical trial	PCL bilayer	Collagen membrane	12 months	24 patients/ 24 surgical areas	-	TG: 0.62 ± 0.38 CG: 0.31 ± 0.19	24 unspecified biological complications
Cucchi et al., 2023 [18]	Randomized clinical trial	d-PTFE reinforced with Ti	Titanium mesh coated with collagen membrane	3 years	40 patients/ 108 surgical areas	TG: 4.26 ± 0.58 CT: 4.42 ± 0.62	-	They report three cases of complications in the test group (one exposure with infection, one abscess without exposure, and one exposure >3 mm without infection) and four cases in the control group (two exposures with infection, one abscess without exposure, and one exposure >3 mm without infection).
Arbab et al., 2016 [19]	Randomized clinical trial	Unreinforced PTFE	Collagen membrane	4 months	24 patients/ 24 surgical areas	TG: −0.5 ± 1.6 CG: −1.2 ± 1.5	TG: −2.2 ± 1.5 CG: −1.4 ± 1.2	No
Deepika et al., 2023 [20]	Randomized clinical trial	PLA-PGA (biomes)	L-PRF membrane	6 months	28 patients/ 28 surgical areas	TG: 3.88 ± 1.56 CG: 3.44 ± 1.21	TG: 1.87 ± 1.57 CG: 2.46 ± 1.38	No
Maiorana et al., 2021 [21]	Prospective clinical trial	d-PTFE	Titanium Mesh (TM)	8 months	5 patients/ 10 surgical areas	TG: 4.2 ± 2.2 CG: 1.5 ± 1.6	-	No in d-PTFE 1 Ti mesh exposure
Shido et al., 2023 [22]	Prospective study	P(LA/CL)	Collagen membrane	5 months (150 days)	20 patients/ 20 surgical areas	-	TG: 4.83 ± 3.3 * CG: 4.27 ± 3.34 *	No
Arunjaroensuk et al., 2018 [23]	Randomized clinical trial	PLA	Collagen membrane	6 months	48 patients 60 surgical areas	-	TG: 2.58 ± 1.08 * CG: 2.89 ± 1.02 *	Three cases with complications in the test group and two in the control group are reported.
Ogata et al., 2021 [24]	A series of cases	P(LA/CL)	No	5 months	5 patients/ 5 surgical areas	-	TG: 2.19 ± 0.62 *	There were no complications except for a fistula reported 30 days later in a single patient, which was eventually resolved.
Li et al., 2023 [25]	Prospective randomized clinical trial	PLA	Collagen membrane	3 years/36 months	48 patients/ 48 surgical areas	-	TG: 2.46 ± 0.34 CG: 2.32 ± 0.46	2 dehiscences with PLA 1 dehiscence with collagen membrane

* Mean bone gain in each measurement relative to the implant. - Means no bone gain data reported.

## Data Availability

The raw data supporting the conclusions of this article will be made available by the authors on request.
